# Stressor exposure has prolonged effects on colonic microbial community structure in *Citrobacter rodentium-*challenged mice

**DOI:** 10.1038/srep45012

**Published:** 2017-03-27

**Authors:** Jeffrey D. Galley, Amy R. Mackos, Vanessa A. Varaljay, Michael T. Bailey

**Affiliations:** 1 Biosciences, College of Dentistry, The Ohio State University, Columbus, OH, USA

## Abstract

Stressor exposure significantly affects the colonic mucosa-associated microbiota, and exacerbates *Citrobacter rodentium-*induced inflammation, effects that can be attenuated with probiotic *Lactobacillus reuteri*. This study assessed the structure of the colonic mucosa-associated microbiota in mice exposed to a social stressor (called social disruption), as well as non-stressed control mice, during challenge with the colonic pathogen *C. rodentium*. Mice were exposed to the social stressor or home cage control conditions for six consecutive days and all mice were challenged with *C. rodentium* immediately following the first exposure to the stressor. In addition, mice received probiotic *L. reuteri*, or vehicle as a control, via oral gavage following each stressor exposure. The stressor-exposed mice had significant differences in microbial community composition compared to non-stressed control mice. This difference was first evident following the six-cycle exposure to the stressor, on Day 6 post-*C. rodentium* challenge, and persisted for up to 19 days after stressor termination. Mice exposed to the stressor had different microbial community composition regardless of whether they were treated with *L. reuteri* or treated with vehicle as a control. These data indicate that stressor exposure affects the colonic microbiota during challenge with *C. rodentium*, and that these effects are long-lasting and not attenuated by probiotic *L. reuteri*.

The human gastrointestinal (GI) tract is the site of many chronic inflammatory illnesses including the inflammatory bowel diseases (IBD), i.e., ulcerative colitis and Crohn’s disease^1^. The exact origins of these illnesses have not been fully explicated. The GI tract has a unique micro-environment that consists of monitoring immune and epithelial cells in close proximity to a constant source of external stimuli and luminal antigen, which can stem in part from the expansive intestinal microbiota that co-exists adjacently^2^. There is normal bidirectional communication between host immune cells sampling the periphery and the microbiota, and disruptions in the microbiota have been associated with negative health outcomes^3^,^4^,^5^. As such, the conditions that skew the composition of luminal antigen or the activity and response of resident host GI cells could be factors that associate with IBD. Psychological stress is one such factor.

Psychological stressor exposure affects GI functioning and symptoms in both healthy and diseased individuals. For example, psychological stress is associated with elevated inflammation, bleeding, and pain in both IBD and enteric infections^6^,^7^,^8^. Although the mechanisms by which psychological stressor exposure leads to heightened inflammatory responses are unknown, previous studies have shown that stressor exposure can affect the GI microbiota in a number of different mammalian hosts, including humans, non-human primates, and rodents^9^,^10^,^11^. Affected bacterial groups included lactic acid bacteria and other health-promoting groups, which were reduced after exposure to stress^11^,^12^. Recently, we have shown that mice exposed to social disruption (SDR), a social stressor that involves aggressive interactions between mice, have significant changes to the mucosa-associated colonic microbiota community structure^10^. The stressor also reduces the absolute abundance of beneficial commensal groups like *Lactobacillus* and *Parabacteroides*. These previous observations were made in healthy, uninfected mice even though studies indicate that stressor-induced changes in the microbiota impact the colonic inflammatory response to *C. rodentium*^6^,^13^,^14^. For example, exposure to stress prior to oral challenge with *C. rodentium* changed gut microbiota composition and increased subsequent colonic inflammatory responses to *C. rodentium*^6^. Transplanting the microbiota from stressor-exposed mice to germfree mice prior to challenge with *C. rodentium* resulted in an exaggerated colonic inflammatory response compared to germfree mice that received microbiota from non-stressed donors^15^, demonstrating the impact that the effects of stress on the microbiota can have on the susceptibility to and severity of *C. rodentium* infection. However, because inflammation in the intestines can significantly change microbial community composition^16^, it is not immediately clear whether stressor-induced changes in gut microbiota composition are still evident in mice with *C. rodentium*-induced colonic inflammation, and whether the effects of the stressor are evident in probiotic-treated animals.

Probiotic bacteria, as defined by the World Health Organization, are living microbes that can confer a health benefit upon a host when given in adequate numbers. *Lactobacillus reuteri* is an immunomodulatory probiotic that can ameliorate the severity of colonic infection^17^ and can down-regulate CCL2, TNF-α, and iNOS mRNA levels in SDR-exposed *C. rodentium*-infected mice, as well as abrogate the heightened colonic pathology in stressor-exposed mice^18^. Probiotic microbes like *L. reuteri* can act directly upon host immunity, such as by modulating phagocytosis and cytokine release by macrophages and monocytes or intestinal epithelial cells^13^,^18^, but they can also affect overall microbiome diversity, which is associated with host health^14^,^19^,^20^,^21^. Thus, it is possible that *L. reuteri* prevents the exacerbating effects of stressor exposure on *C. rodentium*-induced intestinal inflammation by preventing stressor-induced dysbiosis. The purpose of this study was to determine whether the effects of stressor exposure on microbial community composition were evident throughout the course of *C. rodentium* infection and extend beyond termination of the stressor. A secondary objective was to determine whether the effects of the stressor on microbial community composition were evident in probiotic-treated animals.

## Materials and Methods

### Mice

Male C57Bl/6 mice (age 6–8 weeks) were obtained from Charles River (Raleigh, NC), housed three to a cage, and allowed to habituate in an approved Ohio State University vivarium for one week upon arrival. Mice were given food and water *ad libitum* and kept on a 12:12 hour light:dark cycle, with lights on from 0600 to 1800 hr. All procedures were carried out in accordance with guidelines by Office of Laboratory Animal Welfare at the National Institutes of Health and were approved by the Animal Care and Use Committee at the Ohio State University.

### Bacteria

*Citrobacter rodentium*, DBS120, was grown for 18 hr at 37 °C in lysogeny broth. Prior to infection, *C. rodentium* was diluted to a final stock concentration of 3–5 × 10^7^ CFU/mL in PBS. To measure *C. rodentium* in shed stool pellets, stool was homogenized in a slurry in PBS, then plated in serial dilutions in MacConkey Agar with 40 μg/mL of kanamycin added*. Lactobacillus reuteri*, ATCC 23272, was grown for 18 hr at 37 °C at 5% CO_2_ in MRS broth. *Lactobacillus reuteri* was prepared to a stock concentration of 1 × 10^9^ CFU/mL. Each mouse received a total inoculum of 1 × 10^8^ CFU of *L. reuteri*.

### Stress and Infection Study

Test mice were exposed to social disruption stress (SDR), wherein an aggressive CD-1 retired breeder male mouse is placed in a cage with the smaller and younger test mice. The aggressive intruder attacks and defeats the test mice over the course of two hours as previously described^15^,^22^,^23^,^24^. This process is repeated for a total of six evenings, from 1700 to 1900 hr, the beginning of the mouse active cycle. A group termed home cage control (HCC) mice were left undisturbed for the duration of the stressor. The SDR and HCC mice were infected with *C. rodentium (Cr*) immediately following the first cycle of SDR. Each mouse received 100 μl of the *C. rodentium* stock for a total of 3–5 × 10^6^ colony-forming-units (CFU)/mouse. All infected mice had food and water removed for two hours post infection. In addition, following each of the six cycles of SDR, half of the SDR and HCC mice received 1 × 10^8^ CFU of *L. reuteri (Lr*), while the other half of the SDR and HCC mice received PBS vehicle (Veh). In sum, there were four experimental groups: HCC-*Cr*-Veh, HCC-*Cr*-*Lr*, SDR-*Cr*-Veh, and SDR-*Cr*-*Lr*.

### Sacrifice

**Figure 1:**
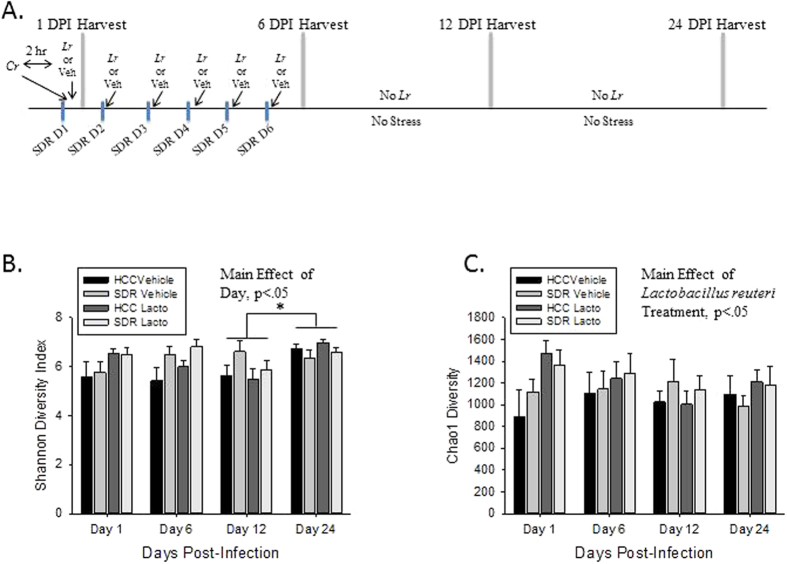
Alpha diversity is affected by probiotic *L. reuteri* treatment and progression of *C. rodentium* infection. (**A**) A timeline of the experimental design. Mice were exposed to SDR from 0 DPI to 5 DPI. All mice were infected w/*C. rodentium* immediately following the first cycle of SDR (0 DPI), and half of the mice were gavaged w/*L. reuteri* following all six cycles of SDR. The other half received a PBS vehicle gavage as control. Mice were harvested at 1, 6, 12, and 24 DPI. (**B**) There is an effect of DPI upon the Shannon Diversity Index. Post-hoc testing indicated that the alpha diversity of mice at 24 DPI were significantly increased over those at 12 DPI. (**C**) There is also an effect of *L. reuteri* treatment upon the Chao1 Richness Index over all DPI.

Mice from the four experimental groups (HCC-*Cr*-Veh, HCC-*Cr-Lr*, SDR-*Cr*-Veh, and SDR-*Cr*-*Lr*) were sacrificed at 1, 6, 12, and 24 days post infection (DPI). Colons were collected for Illumina sequencing analysis, while stool was collected for the purpose of *C. rodentium* quantification. Colons were snap frozen in liquid nitrogen and stored at −80 °C until DNA was isolated for sequencing. An initial experiment was performed, as well as three experimental repeats, for four total experimental runs. The experimental design is shown in Fig. 1A. Total sample sizes at the four time points (1, 6, 12, 24 DPI) varied from 9 to 12 for each experimental group at each time point after combining the four experimental runs. There were a total of 5 uninfected mice, split over two cages, for descriptive comparisons.

### Semi-Quantitative Real-Time PCR

Total RNA was isolated from the distal portion of the colon using Trizol reagent as per manufacturer’s instructions (Invitrogen, Carlsbad, CA), and RNA was reverse transcribed to make complementary DNA using a commercially available kit (Promega, Madison, WI). Real-time PCR primers and probes were synthesized by Applied Biosystems with the sequences as previously reported^8^. Real-time PCR reactions were performed as previously reported^6^. The change in fluorescence was measured using an Applied Biosystems 7000 Sequence Detector and analyzed using Sequence Detector version 1.0 software. In all cases, 18S was used as a housekeeping gene, and the relative amount of transcript was determined using the comparative cycle threshold (C_t_) method as described by the manufacturer.

### DNA Extraction and Library Preparation

DNA was extracted from the proximal section of the colon (~10 mg tissue) using a QIAgen DNA Mini Kit, following manufacturer’s instructions with slight modifications. In summary, colon contents were removed via direct excision, and colon tissues were briefly washed in a PBS bath, so as not to disturb the mucosal layer. Tissues were incubated for 45 mins at 37 °C in lysozyme buffer (20 mg/mL lysozyme, 20 mM TrisHCl, 2 mM EDTA, 1.2% Triton-X, pH 8.0), then bead-beat for 150 sec with 0.7 mm zirconia beads. Samples were incubated at 56 °C for 2 hr with Buffer ATL and Proteinase K, then incubated at 56 °C for 30 mins and 95 °C for 10 mins upon addition of Buffer AL. From this point, the Qiagen DNA Mini Kit isolation protocol was followed from the ethanol step forward. Samples were quantified with the Qubit 2.0 Fluorometer (Life Technologies, Carlsbad, CA) using the dsDNA Broad Range Assay Kit. Samples were standardized to at least 5 ng/μl before being sent to the Molecular and Cellular Imaging Center (MCIC) in Wooster, OH for library preparation. The V1–V3 hypervariable region of the 16S rRNA gene was targeted in this study. To amplify and sequence the V1–V3 hypervariable region of the 16S rRNA gene, we used primers that contain a heterogeneity spacer in line with the targeted sequence. Four sets of spacers of different lengths were used to compensate for the low nucleotide diversity of the amplicons; since accurate base-calling on Illumina platforms and generation of high-quality data requires sequence diversity at each nucleotide position before the clustering occurs. For the targeted region, we used well-known universal primers that were modified to include degenerate bases for maximal inclusiveness^25^.

Libraries were prepared in two rounds of PCR amplification. The first round amplified the locus of interest and added a portion of the Illumina adapter sequence; and the second round completed the Illumina adapter sequence which contained a unique dual combination of the Nextera indices for individual tagging of each sample. Twenty nanograms of each genomic DNA was used as input for the first PCR reaction and 3 μl of the clean PCR 1 product was used as input for PCR 2 reaction. PCR amplifications were carried out as follows: initial denaturation at 96 °C for 3 min, followed by 25 (PCR 1) or 8 (PCR 2) cycles each of 96 °C for 30 sec, 55 °C for 30 sec and 72 °C for 30 sec, and a final extension at 72 °C for 5 min. The PCR products were purified after each PCR amplification using the Agencourt AMPure XP beads (Beckman Coulter Life Sciences, Indianapolis, IN, USA). All the steps for library preparation and cleaning were carried out on the epMotion5075 automated liquid handler (Eppendorf, Hamburg, Germany). The purified amplicon libraries were quantified and pooled at equimolar ratios before sequencing. The final pool was also purified using the Pippin Prep size selection system (Sage Science, Beverly, MA, USA) to discard the presence of any primer dimers.

### Sequencing

The amplicon libraries were sequenced at the Molecular and Cellular Imaging Center (MCIC) in Wooster, OH using the MiSeq sequencing platform (Illumina) at a final concentration of 15.4 pM. A genomic library of well-known diversity previously sequenced in the lab was combined with the pool of amplicon libraries for the sequencing run (expected at 20%). The run was clustered to a density of 1131 k/mm^2^ and the libraries were sequenced using a 300PE MiSeq sequencing kit with the standard Illumina sequencing primers. Image analysis, base calling and data quality assessment were performed on the MiSeq instrument.

### Data Analysis

Forward and reverse ends were demultiplexed using Sabre (website: http://github.com/najoshi/sabre), with 1 allowed barcode mismatch. Barcodes were removed and sequences were trimmed for equal lengths using FastX Trimmer (website: http://hannonlab.cshl.edu/fastx_toolkit). Sequences were joined with Fastq-Join, with 10% allowed differences within the overlap region. Quality filtering was performed with the following parameters: quality score of 20, 0 allowed N characters, 1.5 allowed barcode errors, 3 consecutive low quality bases allowed. qiime_tools (website: http://github.com/smdabdoub/phylotoast) was used for closed reference OTU picking against the 13_8 GreenGenes database^26^. Briefly, the complete dataset was split into smaller.fasta files, and OTUs were picked in parallel on the Ohio Supercomputer using parallel BLAST OTU picking^27^.

### Statistical Analysis

Alpha diversity was measured using the Shannon Diversity index metric, and Chao1 methods. Beta diversity was measured with the unweighted UniFrac distance metric^28^. Alpha and beta diversity were analyzed using Quantitative Insights Into Microbial Ecology (QIIME)^29^. Differences in alpha diversity were calculated with 3 factor ANOVA with DPI, stress group, and probiotic treatment as the between subjects factors, while beta diversity shifts were calculated with adonis, which permutationally analyzes variance in distance matrices^30^. Taxonomic abundances at the phyla and genera levels were normalized by finding the arcsin of the square root of the proportion for each taxonomic classification. The relative abundances were compared using three factor ANOVA with DPI, stress group, and probiotic treatment as the between subjects variables using SPSS v. 21 (IBM, Chicago, IL). Post-hoc LSD tests were used when appropriate. The Benjamini-Hochberg method^31^ was used to correct p values for multiple-tests.

## Results

### Stressor exposure and Infection were both associated with significant alterations to the microbiota

**Figure 2:**
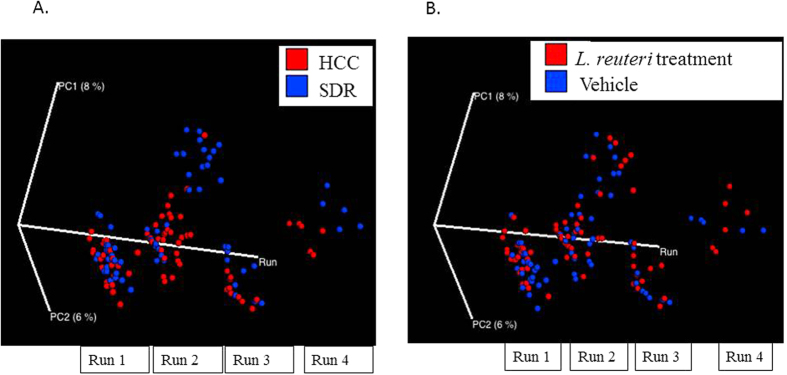
Stressor exposure significantly disturbs microbiota community structure in the overall sample, while probiotic treatment has no effect. (**A**) Mice exposed to SDR had significantly altered microbial profiles, as indicated on a principal coordinate analysis that used unweighted UniFrac distances. This clustering was found to be significant using the adonis statistic. Because the repeated experiments had a significant effect on community structure, the PCoA shows the effect of SDR in each of four repeats of the study on the third axis of the PCoA. (**B**) Probiotic *L. reuteri* treatment was not associated with unique clustering of microbial communities.

Alpha diversity was estimated using the Shannon Diversity Index and Chao1. There was a significant effect of DPI on Shannon Diversity Index (p < 0.05), with post-hoc testing indicating that 24 DPI was higher than 12 DPI (p < 0.05; Fig. 1B). No other DPI were significantly different. When Chao1 was assessed, there was a significant main effect of *L. reuteri* treatment in the 3 factor ANOVA (p < 0.05; Fig. 1C), indicating that *L. reuteri* in general increased Chao1 alpha diversity across the different DPI. Changes in beta diversity of the microbiota community were assessed using the adonis test statistic and the unweighted UniFrac distance metric. When the data were collapsed across all of the experimental runs, the adonis statistic indicated that exposure to the stressor (p = 0.001, R^2^ = 0.01705) and *C. rodentium* infection (p = 0.001, R^2^ = 0.02176) were associated with significant differences in microbial community composition, whereas probiotic gavage with *L. reuteri* did not affect the microbiota structure in the overall community (p = 0.258, R^2^ = 0.00779). However, the adonis statistic also indicated that microbial community composition was significantly different across each of the experimental runs, regardless of treatment condition (p = 0.001, R^2^ = 0.02517). Therefore, in order to visualize the effects of experimental treatments on community composition using a PCoA plot, a custom axis for experimental run was added at the expense of the third PCoA axis. This strategy indicated that exposure to the stressor impacted community composition based on sample clustering in experimental runs 2, 3, and 4 (Fig. 2A), whereas clustering was not present in any of the four experimental runs as a function of probiotic treatment (Fig. 2B).

**Figure 3:**
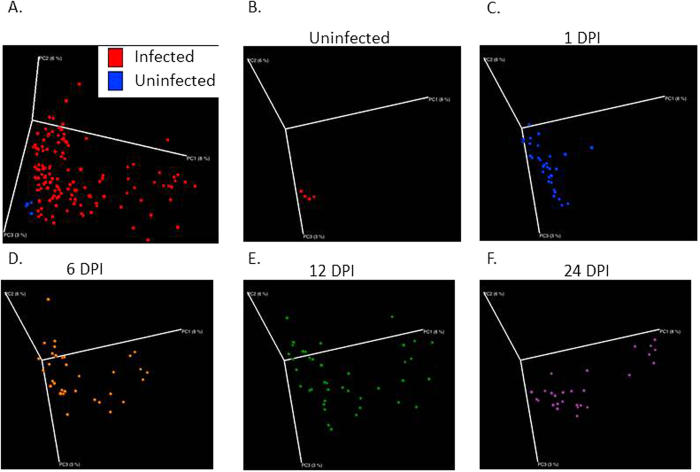
As infection progresses, microbial profiles become increasingly spread along the 3D PCoA space. (**A**) Uninfected mice cluster separately from all infected mice on a principal coordinate analysis (PCoA) based upon unweighted UniFrac distances, which was confirmed with the adonis statistic. (**B–F**) Separating each timepoint indicates dispersion of microbial communities along the first PCoA as the infection continues to 24 DPI.

**Figure 4:**
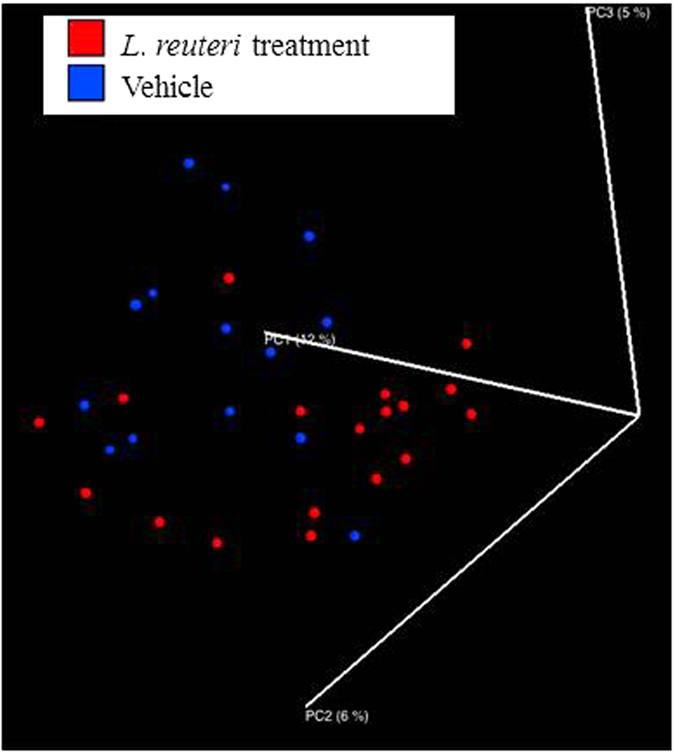
Probiotic treatment significantly shifts the colonic mucosal microbiota at 1 DPI. Unweighted UniFrac distances indicate significant clustering of colons treated with probiotic *L. reuteri*. No later timepoints exhibited clustering based upon probiotic treatment.

**Figure 5:**
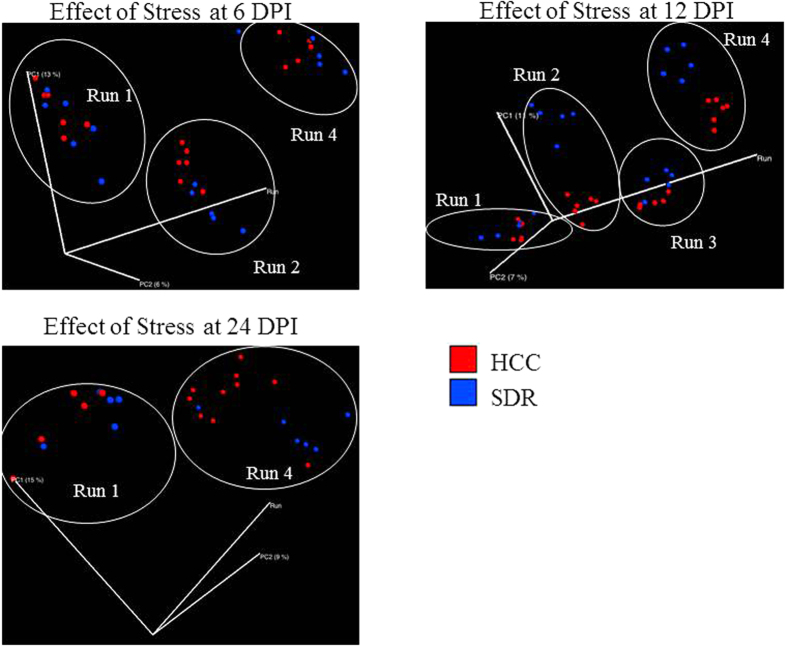
Exposure to SDR affects colonic mucosal microbiota structure regardless of DPI up to 19 days after cessation of exposure. (**A–C**) PCoAs based on unweighted UniFrac were filtered based upon experimental repeat to illustrate the continued effect of stress upon the microbiota at 6, 12, and 24 DPI.

A PCoA produced with QIIME indicated that throughout the time course, the dispersion of the samples increased as a function of DPI (p = 0.001, R^2^ = 0.02405) (Fig. 3). In order to examine how stress and probiotic use affected the microbiota as the infection progressed, samples were filtered based on DPI. At 1 DPI, the *L. reuteri* probiotic had a significant effect upon the structure of the mucosal-associated microbiota (p = 0.005, R^2^ = 0.05243) (Fig. 4), while stressor exposure did not. As the infection proceeded to 6 DPI, the SDR stressor began to impact microbial community composition, as by this point, the mice had undergone six consecutive cycles of the stressor (p = 0.013, R^2^ = 0.0478). Probiotic gavage no longer significantly associated with changes in the microbiota. The effect of the SDR stressor was observed at 12 DPI (p = 0.001, R^2^ = 0.05282) and 24 DPI (p = 0.029, R^2^ = 0.05725) (Fig. 5), despite the cessation of exposure to the stressor. *L. reuteri* treatment did not have an effect at either of these two time points.

### Major taxonomic changes were associated with stress and infection

*Firmicutes* were reduced across the different DPI, as indicated by a significant main effect in the ANOVA (p < 0.001). This main effect was due to significant reductions on 6, 12, and 24 DPI compared with 1 DPI (p < 0.01). In contrast, *Bacteroidetes* were increased across the different DPI (p < 0.001) with significant increases observed on 6, 12, and 24 DPI compared with 1 DPI (p < 0.01). Interestingly, the relative abundances of these phyla were also impacted by stressor exposure. There was a main effect of stress in the ANOVA (p < 0.01) indicating that stressor-exposure significantly reduced the relative abundance of *Firmicutes*. Similarly, stressor exposure significantly affected *Bacteroidetes* (main effect of stress, p < 0.001; Table 1). Stressor exposure did not affect the relative abundance of any other phyla. Moreover, treatment with *L. reuteri* did not significantly affect the relative abundance of any bacterial phyla (Table 1).

At lower taxonomic levels, *S24-7* (p < 0.001) and *Enterobacteriaceae* (p < 0.05) were significantly increased in infected mice, whereas *Prevotella* (p < 0.05) and *Parabacteroides* (p < 0.005) were reduced in those mice. Post-hoc testing indicated that *S24-7* was increased on 6, 12, and 24 DPI compared with 1 DPI (p < 0.001), whereas the *Enterobacteriaceae* were increased on 6 and 12 DPI compared to 1 DPI. *Parabacteroides* levels were reduced on 6 compared to 1 DPI and reduced on 12 DPI compared to 1 and 24 DPI (p < 0.001) (Table 2). *Prevotella* levels were significantly reduced at 12 DPI (p < 0.001) (Table 2).

When the effects of stressor exposure and of *L. reuteri* treatment on the relative abundance of bacterial taxa were tested, it was evident that stressor exposure, but not *L. reuteri* treatment impacted the microbiota (Table 2). Mice exposed to the SDR stressor during *C. rodentium* challenge affected the relative abundance of *Clostridales* (DPI x group interaction, p < 0.05), but this difference did not remain significant after correction for multiple tests. Stressor exposure had a more predominant effect on *S24-7* relative abundance which was significantly increased in SDR-*Cr* mice across the duration of the experiment regardless of whether they were treated with vehicle or with *L. reuteri* (main effect of stress, p < 0.001). *Parabacteroides* and *Lactobacillus* levels were also significantly reduced by stressor exposure throughout the experiment (main effect of stress, p < 0.001), and there was a tendency for *L. reuteri* treatment to normalize *Parabacteroides* (main effect of treatment, p = 0.02 (not significant after correcting for multiple comparisons)) (Table 2).

**Figure 6:**
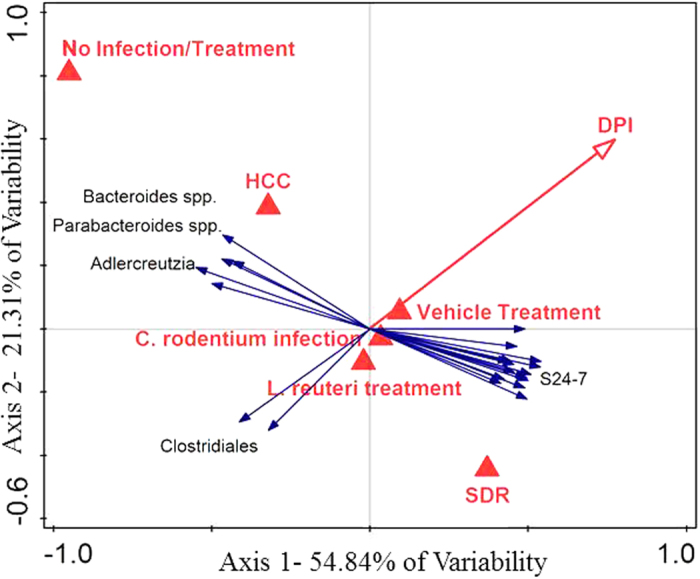
DPI and stressor exposure are associated with specific OTUs on an RDA biplot. OTUs that were identified in *Clostridiales* negatively associated with increasing DPI. OTUs in *Bacteroides* spp., *Parabacteroides* spp., and *Adlercreutzia* spp. associated with HCC and OTUs in *S24-7* associated with SDR. Canoco 5 software was used to construct an RDA plot. OTUs that contributed to at least 20% of the variance were included on the plot.

Redundancy analysis (RDA) using CANOCO further highlighted these results. OTUs with <10 total observations were removed for RDA analysis. DPI (p < 0.01, F = 4.2), Stress (p < 0.01, F = 3.4), and Infection (p < 0.05, F = 1.8) were all significant on the RDA plot. OTUs that accounted for greater than 20% of the variance explained by the two axes were included on the RDA triplot, wherein SDR associated with *S24-7* OTUs, and no stress identified with *Bacteroides, Parabacteroides*, and *Adlercreutzia* OTUs. *Clostridiales* had a negative association with the temporal variable of DPI (Fig. 6).

### Stressor-induced increases in the severity of *C. rodentium* infection were attenuated by probiotic *L. reuteri*

**Figure 7:**
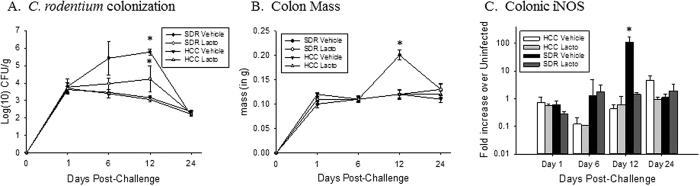
Probiotic *Lactobacillus reuteri* treatment abrogates stressor-induced increase in *C. rodentium*-induced colonic inflammation. (**A**) Mice that were exposed to SDR had significantly higher shed *C. rodentium* in the stool at 12 DPI than control mice. Treatment with *L. reuteri* blocked this increase in *C. rodentium* abundance in the stool. *p < 0.05 vs. HCC Vehicle on Day 12 post-challenge. All groups n = 3 (**B**) Mice exposed to SDR had significantly higher colon mass, an indicator of colitic pathology, at 12 DPI. Mice given *L. reuteri* did not exhibit the same increase in colon mass. *p < 0.05 vs. HCC Vehicle on Day 12 post-challenge. All groups n = 9–12 (**C**) Stressor exposure significantly increased *C. rodentium*-induced colonic iNOS mRNA levels. Treatment with *L. reuteri* attenuated this effect. All groups n = 3. *p < 0.05 vs. all other groups.

Because *L. reuteri* had only a modest effect on alpha diversity and failed to significantly impact beta diversity of the gut microbiota of SDR*-Cr-Lr, C. rodentium* load and markers of colonic inflammation were assessed to verify that exposure to the SDR stressor enhanced the colonic inflammatory response to *C. rodentium*, and that administration of probiotic *L. reuteri* attenuated the effects of the stressor on colonic inflammation as we have previously reported. Consistent with our previous studies, exposure to the stressor significantly increased the amount of *C. rodentium* in the infected mouse stool (Fig. 7A). In the statistical analysis, there was no three-way interaction between Stress, Treatment and DPI, but there was an interaction effect between Stress*DPI (p < 0.05). This indicates that regardless of treatment with probiotic or vehicle, *C. rodentium* levels were significantly increased at 12 DPI in SDR mice over HCC (p < 0.005) (Fig. 7A). Likewise, an interaction effect existed between Stress*Treatment (p = 0.05) indicating that over the course of infection, SDR-*Cr*-Veh mice had significantly more *C. rodentium* than non-stressed HCC-*Cr*-Veh control mice treated with vehicle (p < 0.005). SDR-*Cr-Lr* mice had marginally increased pathogenic burden over HCC-*Cr-Lr* mice that received the probiotic (p = 0.06).

We have previously demonstrated that stressor-induced increases in *C. rodentium* levels are associated with increases in *C. rodentium*-induced colonic pathology, and that *L. reuteri* can attenuate the effects of the stressor on colonic pathology^18^. Thus, markers of colonic inflammation were assessed to verify that the *L. reuteri* attenuated the effects of the stressor in the current study. We previously found that colon mass strongly correlates with colonic histopathology^18^. Thus, we assessed colon mass as a surrogate marker of colonic pathology. Colon mass was significantly increased in mice exposed to the SDR stressor as indicated by a significant interaction between Stress and DPI (p < 0.001). Post-hoc tests indicate that mice exposed to SDR had significantly greater colon mass at 12 DPI (p < 0.005), and marginally increased colon mass at 1 DPI (p = 0.069) and 24 DPI (p = 0.064) (Fig. 7B). There was also an interaction effect between Treatment*DPI (p < 0.001), with post-hoc tests indicating that mice given vehicle treatment had significantly higher colon mass over probiotic-treated mice at 12 DPI (p < 0.005). Similar results were evident when cytokine and inflammatory mediator mRNA levels in the colon were assessed. iNOS levels were significantly higher in mice exposed to the SDR stressor, specifically on 12 DPI (p < 0.0001) (Fig. 7C). This effect was only evident in SDR-*Cr*-Veh mice; stressor-exposed SDR-*Cr-Lr* mice did not have elevations in iNOS mRNA levels in comparison to non-stressed controls on any day post-challenge. Stressor-exposed mice also had increases in TNF-α gene expression on 12 DPI (p < 0.0001); SDR-*Cr-Lr* mice had lower levels of TNF-α than did SDR-*Cr*-Veh mice, but this difference was not statistically significant (data not shown).

## Discussion

Exposure to psychological stressors can alter microbial community structures, turning a normally stable microenvironment into a dysbiotic profile of volatility^9^,^10^,^32^. Disruptions in the microbiota can have serious consequences on host physiology and immunity, and those induced by stress may be associated with aggravation of colonic inflammation. For example, *C. rodentium*-induced colonic inflammation was greater in germfree mice that were colonized with microbiota from donor mice exposed to a prolonged restraint stressor when compared to germfree mice colonized with microbiota from non-stressed control donors^15^. Thus, it is now evident that stressor exposure changes the composition of the gut microbiota that can then lead to exacerbations of colonic inflammation. However, whether changes in the gut microbiota persist and are observed throughout the duration of a colonic inflammatory challenge is not yet known. Thus, we determined whether microbiota community composition was different in stressor-exposed vs. non-stressed mice during ongoing *C. rodentium* challenge.

The current results confirm previous studies demonstrating that the SDR stressor can impact microbiota composition during the 6 day period of stressor exposure^10^,^24^, and extends these previous studies by demonstrating that the stressor effects are long-lasting. Here, the residual impact of the stressor upon the microbiota is evident, as the effect of SDR upon the microbiota can be observed up to 24 DPI (19 days after cessation of SDR). It is possible that the effects of the stressor upon the microbiota at 12 DPI is associated with the infection, because the inflammatory response to the *C. rodentium* challenge peaked at 12 DPI. However, the prolonged effects of the stressor on the microbiota on 24 DPI is not likely due to the *C. rodentium* infection, because *C. rodentium* levels were below the level of detection on 24 DPI and markers of colonic inflammation were not elevated in stressor-exposed mice on 24 DPI. Thus, the effects of the stressor on microbial community composition extended past effects of the stressor on colonic inflammation. This finding has important implications, because many physiological and behavioral effects of the SDR stressor that have been associated with changes to the microbiota are evident up to 14 days after termination of the stressor. For example, both enhanced immune system activity and anxiety-like behavior are evident up to 14 days after termination of the stressor^23^,^33^, and both enhanced immune system activity and anxiety-like behavior have been linked to the microbiota^22^,^34^. Thus, it is possible that the protracted effects of the stressor on microbiota composition contribute to the continuation of immune and behavioral changes after termination of stressor exposure. The long-term relationships between stressor exposure, host physiology and commensal microbes warrant further testing.

Measures of alpha diversity were affected over the 24 day *C. rodentium* challenge (Shannon Diversity) and by *L. reuteri* treatment (Chao1). Alpha diversity was significantly different between 12 and 24 DPI, which overall is the peak of colonic inflammation (12 DPI) and the return to baseline (24 DPI). The effects of *L. reuteri* on alpha diversity were transient, and were only evident on 1 DPI. *L. reuteri* treatment ended on 5 DPI, and by 6 DPI (as well as later time points), Chao1 measures were not different in mice that had been treated with *L. reuteri*. This study also corroborated an earlier study that showed no change in the alpha diversity of the colonic mucosa-associated microbiota as a function of SDR^10^. Interestingly, these findings contrast sharply with previously published research that indicates that chronic restraint stress and water avoidance stress shift the alpha diversity of the colonic mucosa and distal ileum respectively^35^,^36^. Restraint stress and SDR share downstream effects, including increases in anxiety-like behavior in exposed mice. However, considerable psychological and physiological effects differ between the two stressors. Restraint stress is known to induce depressive-like behavior in mice, a hallmark not observed in SDR-exposed mice^37^,^38^. Further, SDR is defined by activation of multiple arms of the immune response, including macrophage oxidative burst and myeloid cell trafficking, while restraint is often associated with immune repression^39^,^40^. Thus, changes to the mucosa-associated microbiota alpha diversity as a function of stress is likely tied to host responses to different types of stressors. Further investigation is required to determine how unique stressors may differentially affect the microbiota.

The RDA plot indicated that the HCC group associates with *Parabacteroides, Adlercreutzia*, and *Bacteroides*, whereas SDR stressor associates with increased *S24-7*. In addition, consistent with previous studies^10^, exposure to the SDR stressor was associated with significant reductions in the relative abundances of *Parabacteroides* and *Lactobacillus*. Both genera have anti-inflammatory properties^8^,^19^,^41^, and their reduction by SDR may be associated with stressor-induced increases in colitis during *C. rodentium* infection. To better understand how SDR-induced disruptions in the microbiota adversely affect host immune responses, the involvement of these microbial groups in immune maintenance and why they are specifically targeted by social stress must be explored in future studies. The importance of *Adlercreutzia* and *Bacteroides* for maintaining health are not completely understood, however some species of *Bacteroides*, namely *B. fragilis*, are known to promote Foxp3+ T regulatory cells that can limit intestinal inflammation^42^. Thus, it is possible that higher levels of *Bacteroides* in non-stressed control mice contribute to increased intestinal homeostasis during pathogen challenge.

Treatment with probiotic *L. reuteri* had little effect on the composition of the gut microbiota. Administering probiotic *L. reuteri* only affected microbial populations at 1 DPI, even though *L. reuteri* administration was repeated through 6 DPI. The repeated *L. reuteri* administration was not sufficient to significantly impact microbial community structure. This result is surprising, because severe inflammation within the GI microenvironment is known to lead to dysbiosis^16^,^43^. This led us to assess whether *L. reuteri* reduced colonic inflammation in animals exposed to the stressor during *C. rodentium* challenge. As with our previous study^18^, probiotic *L. reuteri* significantly attenuated the exacerbating effects of stressor exposure on markers of *C. rodentium*-induced colonic inflammation including colon mass (which we have found closely reflects overall colonic histopathology^18^), and colonic iNOS gene expression. Our finding that *L. reuteri* reduces colonic inflammation, but not microbial community composition, is intriguing, since it is known that inflammation alone can cause changes in microbial community composition^16^. Although it is not yet known why changes in the microbiota were not evident in *L. reuteri*-treated mice, it is possible that stressor exposure leads to changes in microbial composition that are similar to those observed during inflammatory states. This explanation is plausible, since, many stressor-induced changes in microbial community composition, such as changes to *Lactobacillus* and *Parabacteroides*, have also been observed during periods of intestinal inflammation^44^,^45^. Thus, future studies should assess similarities between stressor-induced and inflammation-induced changes in the composition of the gut microbiota.

The current finding that *L. reuteri* reduces colonic inflammation, but not microbial community composition, also suggests that probiotic *L. reuteri* amelioration of pathogen-induced inflammation in stressor exposed animals is not mediated through manipulation of the commensal microbiota. This is an important finding, since several strains of *L. reuteri* have been shown to reduce colonic inflammation^13^,^18^,^46^,^47^,^48^,^49^, but mechanisms by which this occurs are not completely known. Several strains of *L. reuteri* have been shown to reduce cytokine and chemokine production by monocytes, as well as epithelial cells^13^,^18^, thus it is more likely that probiotic *L. reuteri* attenuates pathogen-induced colitis through direct effects on mucosal immune responses rather than mediating an effect through the microbiota. It is also possible that microbial community function, as opposed to microbial community structure, that is truly impacted by *L. reuteri*; others have found that changes in microbial community function can occur with relatively minor changes to microbial community structure^50^. These hypotheses warrant further investigation in future studies.

There is now substantial evidence that the gut microbiota are linked to gastrointestinal illness, such as IBD and irritable bowel syndrome (IBS), as well as systemic diseases, such as diabetes and obesity. And, there are a growing number of studies indicating that the symptoms of many of these same illnesses can be exacerbated during stressful periods. As an example, low social support, perceived distress, the experience of the disease as traumatic, and low quality of life were predictive of disease exacerbation in IBD patients^51^. Moreover, recent, major life events are more common in patients with severe IBD vs. those patients that are in remission^52^. The mechanisms linking stress with exacerbation of these diseases are not completely understood, but the results of this study are consistent with others from our group indicating that stressor-induced changes in the composition of the gut microbiota contribute to dysregulation of mucosal inflammatory responses^15^. Future studies involving assessment of microbial community functions are needed to better understand how stressor-induced alterations of the gut microbiota contribute to dysregulation of colonic inflammation and whether microbial community functions are involved with the beneficial effects of probiotic *L. reuteri* in stressor-exposed animals.

## Additional Information

**How to cite this article**: Galley, J. D. *et al*. Stressor exposure has prolonged effects on colonic microbial community structure in *Citrobacter rodentium*-challenged mice. *Sci. Rep.* **7**, 45012; doi: 10.1038/srep45012 (2017).

**Publisher's note:** Springer Nature remains neutral with regard to jurisdictional claims in published maps and institutional affiliations.

## Figures and Tables

**Table 1:** Phyla Relative Abundances.

	SDR		HCC		SDR		HCC	
	*L.r.*	Veh	*L.r.*	Veh	*L.r.*	Veh	*L.r.*	Veh
	Day 1				Day 6			
Firmicutes*^,†^	85.70 ± 2.86	88.10 ± 2.12	89.96 ± 1.34	78.18 ± 6.27	73.53 ± 6.00	72.21±	80.52 ± 2.46	75.00 ± 5.87
Bacteroidetes*^,†^	9.66 ± 2.02	6.66 ± 1.40	6.56 ± 1.18	8.24 ± 1.98	20.85 ± 6.70	14.14 ± 2.99	10.88 ± 2.25	13.18 ± 3.83
*Proteobacteria*	3.78 ± 0.96	4.62 ± 1.44	2.51 ± 0.54	11.50 ± 4.62	4.75 ± 1.18	10.81 ± 5.77	7.18 ± 2.84	9.98 ± 5.43
*Deferribacteres*	0.56 ± 0.10	0.48 ± 0.22	0.48 ± 0.19	1.79 ± 1.23	0.36 ± 0.10	2.54 ± 1.42	0.96 ± 0.76	1.46 ± 1.18
*Actinobacteria*	0.15 ± 0.04	0.08 ± 0.02	0.30 ± 0.21	0.15 ± 0.09	0.30 ± 0.09	0.20 ± 0.07	0.15 ± 0.03	0.13 ± 0.03
*Verrucomicrobia*	0.06 ± 0.02	0.03 ± 0.02	0.08 ± 0.03	0.03 ± 0.02	0.02 ± 0.02	0.01 ± 0.01	0.21 ± 0.09	0.16 ± 0.12
	Day 12				Day 24			
*Firmicutes*	66.73 ± 6.74	64.54 ± 7.21	76.49 ± 5.24	82.57 ± 2.46	62.27 ± 5.60	72.60 ± 5.99	74.62 ± 6.58	77.37 ± 3.31
*Bacteroidetes*	19.94 ± 3.90	15.94 ± 3.22	8.62 ± 2.65	11.36 ± 2.06	31.20 ± 5.00	18.62 ± 4.91	16.13 ± 6.09	13.22 ± 2.67
*Proteobacteria*	8.04 ± 3.02	17.34 ± 5.38	13.54 ± 4.71	4.43 ± 0.55	4.34 ± 1.12	7.83 ± 2.22	7.17 ± 2.73	7.07 ± 1.45
*Deferribacteres*	0.27 ± 0.10	0.35 ± 0.21	0.85 ± 0.41	0.36 ± 0.09	0.11 ± 0.04	0.17 ± 0.09	0.95 ± 0.45	1.14 ± 0.65
*Actinobacteria*	4.25 ± 2.65	1.41 ± 1.07	0.18 ± 0.07	1.00 ± 0.49	1.28 ± 0.59	0.27 ± 0.14	0.72 ± 0.58	0.70 ± 0.36
*Verrucomicrobia*	0.27 ± 0.11	0.08 ± 0.06	0.25 ± 0.09	0.19 ± 0.10	0.36 ± 0.19	0.02 ± 0.01	0.11 ± 0.05	0.16 ± 0.06

*Indicates a main effect of DPI in the 3 factor ANOVA.

^†^Indicates a main effect of stress in the 3 factor ANOVA.

*L. reuteri* treatment did not affect relative abundances.

**Table 2:** Top 15 Most Abundant Genera.

	SDR		HCC		SDR		HCC	
	*L.r.*	Veh	*L.r.*	Veh	*L.r.*	Veh	*L.r.*	Veh
	Day 1				Day 6			
*Unclassified Clostridiales*	46.86 ± 4.10	37.30 ± 5.36	44.61 ± 4.36	29.00 ± 5.68	42.63 ± 5.10	40.49 ± 5.99	39.00 ± 4.28	29.10 ± 4.21
*Lactobacillus*	18.54 ± 5.35	32.69 ± 8.67	24.12 ± 4.74	33.04 ± 9.82	12.67 ± 3.38	14.23 ± 3.66	24.97 ± 5.66	32.59 ± 7.98
*Lachnospiraceae*	7.96 ± 1.04	7.07 ± 0.80	7.03 ± 0.58	5.50 ± 1.20	7.32 ± 1.20	7.26 ± 1.11	6.61 ± 1.18	4.80 ± 0.72
*S24-7*	0.01 ± 0.01	0.33 ± 0.30	0.05 ± 0.02	2.15 ± 1.45	16.77 ± 6.17	9.58 ± 2.14	2.70 ± 1.50	5.19 ± 2.77
*Bacteroides*	6.01 ± 1.31	3.85 ± 0.67	3.94 ± 0.61	3.42 ± 0.70	1.62 ± 0.32	2.07 ± 0.57	5.49 ± 1.56	3.46 ± 0.96
*Oscillospira*	3.05 ± 0.26	3.26 ± 0.73	3.48 ± 0.34	2.17 ± 0.36	2.87 ± 0.86	3.21 ± 0.61	2.56 ± 0.29	2.47 ± 0.40
*Shewanella*	1.40 ± 0.38	1.76 ± 0.59	0.97 ± 0.20	5.40 ± 2.32	1.35 ± 0.27	4.00 ± 2.42	1.93 ± 0.81	1.69 ± 0.39
*UnclassifiedRuminococcaceae*	2.27 ± 0.25	2.40 ± 0.54	3.36 ± 1.13	1.55 ± 0.26	2.38 ± 0.60	2.10 ± 0.20	1.97 ± 0.32	1.65 ± 0.22
*Lachnospiraceae*; *Ruminococcus*	2.66 ± 0.50	1.37 ± 0.18	2.63 ± 0.58	1.32 ± 0.33	2.25 ± 0.38	2.13 ± 0.48	1.94 ± 0.90	1.94 ± 0.45
*Unclassified Halomonadaceae*	1.20 ± 0.29	1.56 ± 0.48	0.97 ± 0.18	4.29 ± 1.78	1.17 ± 0.19	2.60 ± 1.36	1.45 ± 0.46	1.28 ± 0.23
*Halomonas*	1.10 ± 0.27	1.40 ± 0.39	0.94 ± 0.17	4.04 ± 1.76	1.09 ± 0.16	3.27 ± 2.07	1.39 ± 0.43	1.32 ± 0.33
*Parabacteroides*	1.68 ± 0.44	1.21 ± 0.33	1.51 ± 0.25	1.04 ± 0.20	0.46 ± 0.14	0.33 ± 0.10	1.11 ± 0.17	1.67 ± 0.87
*Prevotella*	1.55 ± 0.43	1.49 ± 0.28	0.93 ± 0.20	1.01 ± 0.25	1.22 ± 0.28	1.05 ± 0.18	1.14 ± 0.27	2.25 ± 0.96
*Enterobacteriaceae*	0.00 ± 0.00	0.01 ± 0.00	0.01 ± 0.00	0.02 ± 0.01	0.60 ± 0.57	0.36 ± 0.24	1.29 ± 1.22	3.01 ± 2.99
*Ruminococcus*	1.13 ± 0.15	1.35 ± 0.39	1.07 ± 0.17	1.02 ± 0.19	0.99 ± 0.25	0.96 ± 0.14	0.92 ± 0.13	0.70 ± 0.11
	Day 12				Day 24			
*Unclassified Clostridiales*	28.46 ± 6.06	32.56 ± 5.06	34.90 ± 5.11	36.20 ± 2.33	27.67 ± 3.54	34.29 ± 8.09	46.57 ± 6.93	44.66 ± 3.19
*Lactobacillus*	20.07 ± 6.75	15.31 ± 5.75	25.51 ± 6.49	25.61 ± 5.70	18.03 ± 2.76	21.98 ± 8.77	10.26 ± 3.58	15.37 ± 2.05
*Lachnospiraceae*	5.22 ± 0.87	7.03 ± 1.14	6.81 ± 0.92	8.13 ± 1.25	4.30 ± 0.60	6.39 ± 0.66	7.08 ± 1.08	6.24 ± 0.54
*S24-7*	12.05 ± 2.60	8.37 ± 2.80	2.31 ± 1.55	4.99 ± 1.92	18.76 ± 4.68	11.44 ± 3.93	8.24 ± 5.62	0.98 ± 0.44
*Bacteroides*	1.59 ± 0.28	3.49 ± 1.62	3.82 ± 0.98	3.78 ± 1.29	6.23 ± 2.07	2.81 ± 1.34	3.62 ± 1.44	6.47 ± 1.46
*Oscillospira*	2.47 ± 0.56	2.67 ± 0.48	2.59 ± 0.36	2.56 ± 0.33	2.23 ± 0.22	2.02 ± 0.48	2.68 ± 0.36	2.76 ± 0.34
*Shewanella*	1.75 ± 0.50	4.36 ± 1.90	2.09 ± 0.46	1.30 ± 0.15	1.44 ± 0.43	2.82 ± 0.82	2.70 ± 1.17	2.64 ± 0.52
*UnclassifiedRuminococcaceae*	2.22 ± 0.51	2.23 ± 0.26	1.78 ± 0.25	1.90 ± 0.28	1.45 ± 0.23	2.35 ± 0.74	2.53 ± 0.47	2.97 ± 0.55
*Lachnospiraceae; Ruminococcus*	2.23 ± 0.71	1.72 ± 0.39	1.44 ± 0.21	2.07 ± 0.37	1.86 ± 0.27	1.55 ± 0.14	1.74 ± 0.19	1.95 ± 0.35
*Unclassified Halomonadaceae*	1.34 ± 0.30	3.68 ± 1.50	1.69 ± 0.32	1.12 ± 0.13	1.37 ± 0.36	2.19 ± 0.57	1.83 ± 0.59	2.23 ± 0.47
*Halomonas*	1.42 ± 0.34	3.47 ± 1.50	1.54 ± 0.30	1.08 ± 0.10	1.22 ± 0.32	2.45 ± 0.87	2.06 ± 0.83	2.13 ± 0.46
*Parabacteroides*	1.21 ± 0.43	0.47 ± 0.09	1.43 ± 0.37	1.44 ± 0.42	0.92 ± 0.16	0.99 ± 0.20	1.42 ± 0.46	3.21 ± 0.77
*Prevotella*	0.74 ± 0.28	1.06 ± 0.41	0.63 ± 0.16	0.51 ± 0.13	1.48 ± 0.47	1.50 ± 0.47	0.87 ± 0.23	1.85 ± 0.53
*Enterobacteriaceae*	1.84 ± 1.74	3.01 ± 2.14	4.50 ± 2.84	0.49 ± 0.20	0.03 ± 0.01	0.04 ± 0.04	0.24 ± 0.20	0.01 ± 0.00
*Ruminococcus*	0.74 ± 0.15	0.81 ± 0.16	0.93 ± 0.13	0.88 ± 0.11	0.68 ± 0.10	0.83 ± 0.22	1.08 ± 0.19	1.12 ± 0.09
